# A review of the foot function index and the foot function index – revised

**DOI:** 10.1186/1757-1146-6-5

**Published:** 2013-02-01

**Authors:** Elly Budiman-Mak, Kendon J Conrad, Jessica Mazza, Rodney M Stuck

**Affiliations:** 1 Center for Management of Complex Chronic Care, Staff Physician, Medical Service, Hines, VA Hospital, 5000 South 5th Ave, Hines, IL, 60141-3030, USA; 2 Department of Medicine Loyola University Stritch School of Medicine, Loyola University of Chicago, Maywood, IL, 60513, USA; 3 Health Policy and Administration (MC 923) School of Public Health University of Illinois at Chicago, 1603 West Taylor Street, Chicago, IL, 60612-4394, USA; 4 University of Illinois at Chicago School of Public Health (MC923), 1603 West Taylor Street, Chicago, Illinois, 60612, USA; 5 Department of Orthopaedic Surgery, Loyola University Stritch School of Medicine, Loyola University of Chicago, 2160 South First Ave, Maywood, IL, 60153, USA; 6 Surgical Service, Hines VA Hospital, 5000 South 5th Ave, Hines, IL, 60141-3030, USA

**Keywords:** FFI, FFI-R, FFI adaptation/translation, FFI scores, Foot health measures

## Abstract

**Background:**

The Foot Function Index (FFI) is a self-report, foot-specific instrument measuring pain and disability and has been widely used to measure foot health for over twenty years. A revised FFI (FFI-R) was developed in response to criticism of the FFI. The purpose of this review was to assess the uses of FFI and FFI-R as were reported in medical and surgical literature and address the suggestions found in the literature to improve the metrics of FFI-R.

**Methods:**

A systematic literature search of PubMed/Medline and Embase databases from October 1991 through December 2010 comprised the main sources of literature. To enrich the bibliography, the search was extended to BioMedLib and Scopus search engines and manual search methods. Search terms included FFI, FFI scores, FFI-R. Requirements included abstracts/full length articles, English-language publications, and articles containing the term "foot complaints/problems." Articles selected were scrutinized; EBM abstracted data from literature and collected into tables designed for this review. EBM analyzed tables, KJC, JM, RMS reviewed and confirmed table contents. KJC and JM reanalyzed the original database of FFI-R to improve metrics.

**Results:**

Seventy-eight articles qualified for this review, abstracts were compiled into 12 tables. FFI and FFI-R were used in studies of foot and ankle disorders in 4700 people worldwide. FFI Full scale or the Subscales and FFI-R were used as outcome measures in various studies; new instruments were developed based on FFI subscales. FFI Full scale was adapted/translated into other cultures. FFI and FFI-R psychometric properties are reported in this review. Reanalysis of FFI-R subscales' confirmed unidimensionality, and the FFI-R questionnaires' response categories were edited into four responses for ease of use.

**Conclusion:**

This review was limited to articles published in English in the past twenty years. FFI is used extensively worldwide; this instrument pioneered a quantifiable measure of foot health, and thus has shifted the paradigm of outcome measure to subjective, patient-centered, valid, reliable and responsive hard data endpoints. Edited FFI-R into four response categories will enhance its user friendliness for measuring foot health.

## Background

Foot problems commonly arise during our daily living activities [1,2]. The prevalence of foot problems in general ranges between 10% and 24% [3]. Their prevalence is higher among older individuals and in chronic rheumatoid arthritis (RA), gout, and diabetes mellitus with peripheral neuropathy [4]. Foot pain and disability can affect workers’ productivity, work absenteeism, and other issues [5,6]. Because pain and disability are subjective complaints, they are difficult to quantify without a valid patient report of the degree to which an individual is experiencing foot pain. Without a valid measure, problems arise in documenting foot health status, tracking the progression of diseases, and establishing the efficacy of treatment, including assessment of treatment satisfaction and of health related quality of life from a personal perspective.

In 1991, the Foot Function Index (FFI) was developed as a self-reporting measure that assesses multiple dimensions of foot function on the basis of patient-centered values. The FFI consists of 23 items divided into 3 subscales that quantify the impact of foot pathology on pain, disability, and activity limitation in patients with RA [7]. The FFI was developed using the classical test theory (CTT) [8] method. It has been found to have good reliability and validity and has had wide appeal to clinicians and research scientists alike [3,9,10]. In the past 20 years, the FFI has been widely used by clinicians and investigators to measure pain and disability in various foot and ankle disorders and its use has expanded to involve children, adults, and older individuals. Furthermore, the FFI has been widely used in the study of various pathologies and treatments pertaining to foot and ankle problems such as congenital, acute and chronic diseases, injuries, and surgical corrections.

In 2006, the FFI was revised (the FFI-R) on the basis of criticisms from researchers and clinicians; items were added, including a scale to measure psychosocial activities and quality of life related to foot health [11].

A literature review was conducted to develop a theoretical model of foot functioning [12], based on the World Health Organization International Classification of Functioning (ICF) model. The FFI-R items were developed from the original 23 FFI items, and more items were added as a result of the literature review. As a result of clinicians and patients’ input, the final draft of the FFI-R, which consisted of 4 subscales and 68 items, was completed. The results were the FFI-R long form (FFI-R L; 4 subscales and 68 items) and the FFI-R short form (FFI-R S; 34 items) as total foot function assessment instruments. Both the 68-item and 34-item measures demonstrated good psychometric properties.

The FFI-R in its current form is one of the most comprehensive instruments available. However, in a review article [13], questions were raised about the unidimensionality and independence of FFI-R subscales, and we did not include such reports in our previous article about the FFI-R [11]. We carefully reviewed the comments about the FFI-R and assessed the unidimensionality of the subscales by use of the Rasch model. On the basis of these critiques, the FFI-R required a periodic revision of its metrics to ensure it represented patient-centered health values and state-of-the-art methodology.

Our aim is to assess the contribution of the FFI and FFI-R to the measurement of foot health in the fields of rheumatology, podiatry, and orthopedic medicine. This assessment should enable us to reflect on and improve the quality of the measure. Therefore, we conducted a systematic review of literature pertaining to the FFI and FFI-R that has been published in the English language from October 1991 through December 2010. The objectives were to: (i), Assess the prevalence of uses of the FFI and FFI-R in clinical studies of foot and ankle disorders; (ii), Describe the utility and clinimetric properties of the FFI and FFI-R as they have been applied in various clinical and research settings; (iii), Enumerate the strengths and weaknesses of the FFI and FFI-R as reported in the literature; (iv), Address the suggestions found in the literature for improving the FFI-R metrics.

## Methods for systematic search of the literature

This study was about a systematic review of articles in which the FFI and/or FFI-R were used as measures of a variety of foot and ankle problems. Relevant studies were identified by English language publication searches of the electronic bibliographic databases Pub Med/MEDLINE, EMBASE, BioMedLib and Scopus from October 1991 through December 2010.

### Search terms and eligibility criteria

The key words: *foot function index, FFI scores, foot function index scores,* and *foot function index revised (FFI-R).*were used as search terms and was applied to all databases. *FFI instruments/measure* and/or *FFI-R instruments/measure* had to be mentioned in the abstracts and in the full articles to be collected for in-depth scrutiny. Articles fulfilling the inclusion criteria were selected for the review. The article criteria included: (i) the words *foot function index/FFI* or *revised foot function index/FFI-R* in its reports/measures; (ii) full-length articles; (iii) written in English and published from October 1991 through December 2010; (iv) the study population described needed to have foot complaint(s)/problems; and (v) regardless of the country conducting the study, the full-length article must have been published in English or in a foreign language with the abstract in English.

### Objectives with method of data collection and organization of tables

Selected articles that fulfilled the criteria were independently reviewed and collected by the authors to address the objectives and organize collected data into several tables.

### Objective 1. Uses of the FFI and FFI-R

We created four tables to address the first objective of describing the measurement’s uses (Tables 1, 2, 3, and 4).

**Table 1:** Study type, sample size and sample characteristics

**Study type**	**Number**	**Sample size (N)**	**N Male**	**N Female**	**Age (SD)**
Measurement	17	1236	458	763	54.9 (6.4)
Surgery	30	1512	648	857	45.1 (15.7)
Orthoses	19	1101	493	521	43.0 (15)
Other intervention	4	170	55	115	47.6 (6.1)
Observational	8	695	260	432	52.2 (27.9)
Total	78	4714	1914* (41%)	2688* (57%)	48.58 (4.9)

*Gender not reported in 3 studies: Slattery, M [82] (2001), Clark, H [85] (2010) and Kulig, K [88] (2009).

**Table 2:** FFI uses across studies in foot and ankle disorders including diagnoses

**Diagnosis**	**Measure**	**Surgery**	**Orthosis**	**Observational**	**Other**	**Total**
Rheumatoid arthritis	6	5	7	3		21
Osteoarthritis	2	1		1		4
Juvenile arthritis			1			1
Hallux valgus	2	2	1			5
Hallux rigidus		3				3
Plantar fasciitis/heel pain	2	2	4		3	11
Metatarso phalangeal arthritis		2	2			4
Chronic foot pain	3	2		1		6
Foot and ankle fracture	1	5	1**	1		8
Posterior tibial tendon pain			1		1	2
Bone graft		1				1
Ankle deformity		2				2
Flat foot		1				1
Cavovarus Charcot-Marie-Tooth		2				2
Osteo-chondral lesion of talus-tibia		1				1
Failed total ankle arthrodesis		1				1
Club foot		1				1
Diabetic neuropathy				1		1
Mid foot pain	1		2			3
Paget disease				1		1
Total	17	31*	19	8	4	79*

*Two different diagnoses occurred in one study, **Hemophilic ankle arthropathy.

**Table 3:** FFI Uses across studies conducted internationally

**Country**	**Measure**	**Surgery**	**Orthosis**	**Observational**	**Other**	**Total**
Australia	2	1	1			4
Austria		2				2
Brazil			2			2
Canada		2		1		3
Czech Rep.		2				2
France		1				1
Germany	1	1		2	1	5
Japan		1				1
So. Korea			1			1
Netherlands	2	7				9
New Zealand			1			1
Slovenia			1	1		2
Sweden		1				1
Taiwan	1					1
Turkey	1			2		3
UK	2	1	3	2		8
USA	8	12	9		3	32
**Total**	17	31	18	8	4	**78**

**Table 4:** FFI Full scale and subscale used across studies

**FFI**	**Measure**	**Surgery**	**Orthosis**	**Observational**	**Other**	**Total**
FFI Full scale (3 domains)	10	21	14	6		51
FFI Pain scale	2	1	2	2	3	10
FFI Disability scale		1				1
FFI Pain and Disability scale	3	3	1		1	8
FFI - 5pts	1	4				5
FFI-R Long form	1		2			3
FFI Used in studies	17	30	19	8	4	78

### Objective 2. Utility and clinimetric properties

We designed a data-collection form to address the second objective. This form was assessed in a pilot study by collecting data from ten articles out of the collection of qualified articles; it was revised before being used in its current format. The variables used in this data-collection form were: (i) the instrument and year the article was published; (ii) the first author’s name; (iii) the objectives of the study; (iv) the population characteristics, sample size, and diagnosis; (v) psychometric analysis (reliability and validity, etc.); (vi) items/domains/subscales of the FFI or FFI-R used in the study; (vii) response type; and, (viii) a short summary evaluation of each study. Therefore, this data form recorded the analytic statements extracted from each article, and 6 tables were created (Tables 5, 6, 7, 8, 9, and 10). Data were arranged in each table in chronological order.

**Table 5:** Studies of foot function measures

**Instrument**	**1**^**st **^**Author**	**Objective**	**Population *(N, Sex, Age, Dx, location)***	**Psychometric analysis**	**Items/domains/subscales/item sources**	**Response type**	**Summary evaluation**
Foot Function Index, 1991	Budiman-Mak, E [7]	Instrument Development	N: 87 (78 male)	Classical Test Theory	23 items	Visual Analog Scale	Good clinimetrics, applicable to various age groups and varieties of foot and ankle pathologies.
			Mean age: 61		3 domains		Conclusion: Positive
			(Range: 24–79)		Pain, difficulty and activity limitation subscales clinician		
			Dx: RA foot				
			Location: USA				
Foot Function Index Pain (left/right), 1996	Saag, KG [23]	Foot Function Index pain scale; Compare right/left foot	N: 63 (13 male)	Classical Test Theory	9 items	Visual Analog Scale	This measure of right vs. left side of the foot showed good clinimetric properties
			Mean age: 57.5 (SD=11.6)		FFI pain subscale		Conclusion: Positive.
			Dx: RA		clinician		
			Location: USA				
Foot Function Index/Foot Health Status Questionnaires (FHSQ), 1998	Bennet PJ [9]	Development of FHSQ, a new measure	N: 111 (25 male)	Classical Test Theory	13 items	Likert	FHSQ has good clinimetrics.
			Mean age: 54 (SD=20)		4 domains clinician		Conclusion: Positive.
			Dx: Osteoarthritis hallux valgus				
			Location: Australia				
Foot Function Index/Ankle Osteoartitis Score (AOS), 1998	Domsic, RT [24]	AOS consisted of Foot Function Index pain and disability scales	N: 36 (12 male)	Classical Test Theory	18 items	Visual Analog Scale	AOS had good clinimetrics.
			Mean age: 52.7 (Range: 16–79)		2 Domains clinician		Conclusion: Positive.
			Dx: Ankle osteo-arthritis				
			Location: USA				
Foot Function Index/Foot Function Index- 5pts in Dutch, 2002	Kuyvenhoven, MM [3]	Foot Function Index in Dutch	N: 206 (78 male)	Classical Test Theory	15 items	5-point Likert	Adaptation of Foot Function Index to 5 point Likert, used as a generic measure in foot and ankle problems.
			Mean age: 61 (SD=10)		2 domains: pain & disability clinician		Conclusion: Positive.
			Dx: OA with limited mobility and pain				
			Location: Netherlands				
Foot Function Index/Foot Health Status Questionnaire (FHSQ), 2002	Landorf, KB [10]	Validation of FHSQ to Foot Function Index	N: 17 (4 male)	Non-parametric statistics	FHSQ	5-point Likert	FHSQ has less items than FFI and was printed in larger font for ease of use.
			Mean age: 44.6 (SD=10.5) (Range 24–72)		13 items		Conclusion: Positive.
			Dx: Painful plantar fasciitis		4 domains; clinician		
			Location: Australia				
Foot Function Index/Foot Impact Scale (FIS), 2005	Helliwell, P [29]	Validation with Health Assessment Questionnaire (HAQ), FFI, and Manchester Foot Disability Questionnaires (MFDQ)	N: 148 (34 male)	Item Response Theory	FIS	Visual Analog Scale	FIS items were derived from RA patients (consisted of impairment/shoes and activities/participation subscales), with good clinimetric properties.
			Mean age: 61.7 (Range 28–89)		51 items		Conclusion: Positive.
			Dx: RA Foot Pain		2 domains		
			Location: UK		Patient		
Foot Function Index, 2005	Agel, J [25]	Reliability and validity tests in specific population with moderate to high physical function	N: 54 (22 male, 6 unknown)	Correlation statistics	Foot Function Index	Likert Scale	Use of Foot Function Index in non-systemic foot and ankle problems requires removal of 2 items each from pain and disability, judged not applicable for this condition.
			Mean age: 52.8 (SD=12.3) (Range 19–74)		23 items		Conclusion: Positive.
			Dx: Non-traumatic foot/ankle complaints		3 domains		
			Location: USA				
Foot Function Index, 2005	Shrader, JA [28]	Reliability and validity measures of navicular joint deformity vs. clinical findings	N: 20 (0 male)		Foot Function Index	Visual Analog Scale	Foot Function Index was used to measure the foot health status associated with joint deformities.
			Mean age: 55.4 (SD=11.4 years); Dx: RA 12.7 years (SD=10.4)		Index 23 items		Conclusion: Positive.
			Dx: Navicular joint dropped and foot pain		3 domains		
			Location: USA				
Foot Function Index-R with Foot Function Index, 2006	Budiman-Mak, E [11]	Instrument Development	N: 97 (90 male)	Item Response Theory	Foot Function Index	Likert scale (replaced Visual Analog Scale)	Foot Function Index-R had 3 domains, plus 4^th^ psychosocial domain added to assess quality of life.
			Mean age: 69 (range: 38–88)		68 items (long)		Conclusion: Positive
			Dx: Chronic foot and ankle complain		34 items (short)		
			Location: USA		Clinicians and patients		
Foot Function Index, 2006	Bal, A [26]	Comparing Foot Function Index with Health Assessment Questionnaires (HAQ) & SFC	N: 78 (11 male)	Correlation statistics	Foot Function Index	Visual Analog Scale	Strong correlations of HAQ and Foot Function Index scores, HR and CV also reflected in Foot Function Index scores and were highly correlated with Rand 36 items Short Form Health Survey (SF36).
			Mean age: 50.65 (SD=10.7); RA duration 13.96 (SD=8.09)		23 items		Conclusion: Positive
			Location: Turkey		3 Domains		
		
Foot Function Index & SF36, 2006	SooHoo, N [27]	Validity test in foot health and general physical health	N:69 (25 male)	Correlation statistics	Foot Function Index	Visual Analog Scale	The 3 domains of Foot Function Index demonstrated moderate-high correlation with SF36, thus it was reasonable to use Foot Function Index to monitor outcomes.
			Mean Age: 46 (Range 16–82)		23 items		Conclusion: Positive.
			Dx: Foot & Ankle disorder		3 domains		
			Location: USA				
Foot Function Index & American Orthopedic Foot and Ankle Society (AOFAS) hallux module, 2006	Baumhauer, JF [32]	Reliability and validity of test, compared with Foot Function Index	N:11 (1 male)	Correlation statistics	AOFAS hallux & lesser toes module	Numeric rating scale	Only AOFAS hallux for pain correlated with Foot Function Index pain scale.
			Mean age: 54 (Range: 40–72)				Conclusion: Positive.
			Dx: RA without foot complaints				
			Location: USA				
Foot Function Index, 2006	Van der Leeden, M [30]	Measure forefoot damage	N:62 (15 male)	Correlation Statistics	Validation with Western Ontario Mac Masters Universities Osteoarthritis Index (WOMAC) and Disease Activity in 44 RA joints (DAS-44)	Numeric rating scale	Foot Function Index function subscale correlated with WOMAC and DAS-44. Foot Function Index pain score correlated with forefoot pain. Foot Function Index function score correlated with hind foot problem.
			Mean age: 55.7 (SD=13.11)				Conclusion: Positive.
			Dx: RA forefoot complaints, duration of 96 months				
			Location: Netherlands				
Foot Function Index, American Orthopedic Foot and Ankle Society (AOFAS) clinical rating component, 2007	Ibrahim, T [33]	Testing the criterion validity of clinical rating components of AOFAS with Foot Function Index	N:45 (11 male)	Correlation Statistics	Validity of AOFAS scale	Numeric rating scale	The scores of AOFAS clinical ratings and Foot Function Index were moderately correlated based on 41% response rate.
			Mean age: 55 years (range=15-81)				Conclusion: Positive.
			Dx: Hallux deformities				
			Location: UK				
Foot Function Index,/Foot Function Index Chinese (Taiwan), 2008	WU, SH [36]	Reliability and validity measure of PCS of SF-26, Taiwan version;	N:50 (planta fasciitis); mean age 46.9 (SD=10.6)	Cross-cultural adaptation	Foot Function Index	Visual Analog Scale	Foot Function Index Taiwan Chinese consisted of 21 items. Could measure non-traumatic and traumatic foot and ankle problems. The floor score was 10%, in sample with fractures.
			N:29 (ankle/foot fracture); mean age 37.2 (SD=14.8) 25 male		21 items		Conclusion: Positive.
			Location: Taiwan		3 domains		
					The order of items was changed.		
					Clinician and patient		
Foot Function Index, Foot Function Index-D, 2008	Naal, FD [34]	Foot Function Index-D,	N:53 (14 male)	Cross-cultural adaptation	Foot Function Index-D	Numeric rating scale	Foot Function Index underwent German translation. Foot Function Index-D added 3 new items and revised 8 items of the Foot Function Index and had demonstrated good clinimetrics.
			Age: 57.2 (SD=13.7) Range (18=77)		Index-D 18 items (pain & disability subscales)		Conclusion: Positive.
			Dx: Foot complaints		2 domains		
			Location: Germany		Clinician and patient		

**Table 6:** Clinimetric properties of patient-reported foot function measures

**Instrument; author year**	**Reliability e.g., IRT, CTT ICC, kappa, test-retest**	**Cronbach’s alpha**	**Instrument /Domain N items/ Item generated sources **	**Validity (Face, content, criterion or construct) and other measures**	**Response to change**	**Completion time**	**Sample N diagnoses conclusion**
FFI; Budiman Mak, E [7] 1991	CTT	Total: 0.96	FFI	Face: yes	Yes	10 minutes	N=87
	ICC total: 0.87	Pain: 0.70	23 items	Criterion: r=0.52 FFI total scores vs 50 ft walked			Early rheumatoid arthritis
	ICC (pain): 0.70	Disability: 0.93 Activity	Clinician and patient	Construct: Yes			Conclusion: Positive
	ICC (disability): 0.84	Limitation 0.73					
	ICC (activity limitation): 0.81						
FFI pain subscale (R/L foot); Saag, KG [23] 1996	CTT	0.94-0.96	FFI side-to-side; Clinician and patient	Face: Yes			N=63 Rheumatoid foot pain
	ICC: 0.79-0.89			Content: Yes			Conclusion: Positive
FFI and AOS; Domsic, RT [24]1998	CTT		AOS	Criterion: AOS vs WOMAC disability			N=562
	ICC: 0.97		18 items; Clinician	r=0.65 pain r=0.79			Dx: Ankle Osteoarthritis
	Pain: 0.95			Construct: Yes			Conclusion: Positive
	Disability: 0.94						
FFI & FHSQ. Bennet, PJ [9]1998	CTT	0.85-0.88	FHSQ	Criterion: Yes		3-5 minutes	N=255 Dx: Hallux valgus osteoarthritis
	ICC	Pain: 0.88	13 items	Construct: Yes, discriminant validity; Goodness of Fit			Conclusion: Positive
	0.74-0.92	Function: 0.85	Clinician and Patient				
	pain 0.86	Footwear: 0.85					
	function 0.92	Foothealth: 0.87					
	footwear 0.74						
	foothealth 0.78						
FFI (5 pt); Kuyvenhoven, MM [3] 2002	CTT	0.88-0.94	FFI (5 pt)	Concurrent validity: Yes	Yes		N=206
	ICC 0.64-0.79	Total: 0.93	15 items				Dx: Non-traumatic foot complaint
	Total: 0.76	Pain: 0.88	Clinician				Conclusion: Positive
	Pain: 0.64	Disability: 0.92					
	Disability: 0.79						
FFI & FIS; Helliwell,P [29] 2005	IRT	Not performed	FIS	Face: Yes			N=192
	ICC		51 items	Content: Yes			Rheumatoid arthritis
	Impairment/shoes: 0.84 Activities/participation: 0/96		2 subscales	Construct: Yes			Conclusion: Positive
			clinician and patient	Goodness of Fit			
						
FFI; Agel, J [25] 2005	ICC		FFI				N =54 FFI was tested in non-systemic or traumatic foot problems.
	Total: 0.68		19 items items each from pain and difficulty subscales were deleted				FFI was good for individuals with low level functioning.
	All subscale values were significant at .01 level		Clinician				Conclusion: Positive
FFI-R; Budiman-Mak, E [11] 2006	IRT	Total: 0.95	FFI-R	Criterion: Yes		15 minutes	N=92
	Person reliability: 0.96	Pain: 0.93	Long form (68 items); Short form (34 items) Clinician and patient	Construct: Yes			Dx: Chronic foot and ankle problems
	Item reliability:0.93	Disability: 0.93		Minimal floor effect (4.5%)			Conclusion: Positive
		Activity limitation: 0.88		Goodness of Fit			
		Psychosocial: 0.86					
FFI & SF 36: SooHoo, NF [27] 2006	Pearson Correlation of FFI to SF-36: Pain: -0.10 to −0.61;		FFI	Construct: Yes			N=69
	Disability: -0.23 to −0.69		23 items				Forefoot and hindfoot complaints
	Activity limitation: -0.23 to −0.61		3 domains				Moderate correlation between FFI and SF-36
							Conclusion: Positive
FFI AOFAS; Baumhaur, JF [32] 2006	ICC AOFAS Summary Scores: Hallux 0.95 Lesser toes: 0.8 Pearson’s correlations mean value AOFAS Hallux vs. FFI: r=0.80, AOFAS lesser toes vs FFI: r=0.69; Pain subscale AOFAS Hallux vs. FFI summary score: r=0.31		FFI	Content: Yes			N=11
			23 items	Criterion: Yes			Rheumatoid Hallux and lesser toes
			3 domains	Ceiling effect noted in lesser toe activity subscale			Conclusion: Positive
FFI FHSQ ; Landorf, KB [101] 2007	ICC measures were reported; Minimal important difference (MID) was the focus of this clinical measure		MID				N=175
			FHSQ Pain 14, Function 7, General health 9				Plantar fasciitis
			FFI Pain 12, Function 7, Total 7				Conclusion: Positive
			VAS				
			Pain 9				
FFI, AOFAS; Ibrahim, T [33] 2007	Test-retest AOFAS; pre and post operation was no different; 41% response rate. Pearson correlation with FFI was −0.68 for all the subjective components of AOFAS. Hallux module subjective component was −0.46		AOFAS subjective component; Items dependent on modules	Criterion: yes	Yes		N=45 Foot and ankle problems
			Clinician	Construct: Yes			AOFAS reliability and validity was tested.
				Discriminant and predictive validity			Conclusion: positive with caution due to several limitations as mentioned in the paper.
FFI, FFI Taiwan Chinese; Wu, SH [36] 2008	ICC	CA		Criterion: Yes Floor effect 10%			N=79
	Total 0.82	Total 0.94					Traumatic (fracture) non-traumatic plantar fasciitis foot problems
	Pain 0.74	Pain 0.91					Conclusion: positive with caution, due to limitations (see article)
	Disability 0.76 activity limitation 0.88	Disability 0.95					
		Activity limitation 0.75	Clinician and patients				
		Pearson’s correlations					
		FFI total with SF 36 r=−0.59 plan- tar fasciitis r=−0.61 ankle fracture					
FFI, FFI- German Naal FD [34] 2008	ICC	CA total 0.97	FFI German 18 items pain and disability subscales 3 items were added to the instrument by patients	Construct yes Convergent validity FFI-G vs PCS of SF-36, VAS pain, disability UCLA activity scale	Yes	8.3 min	N= 53
	Total 0.98	pain 0.90	Clinician and patients				Various foot problems required surgery
	Pain 0.97	disability 0.95	Patient related difficulty 2.4 of rating scale 1-10				
	Disability 0.99	Cross cultural adaptation English to German with forward and backward protocol					Conclusion: positive
FFI-R; Rao S [75] 2009	This report is about minimal detectible change (MDC_90_) a measure of clinical importance.		FFI-R long 68 items	MDC Total 5 Pain 5			N=22 Orthoses treatment in mid foot pain
	A result of orthoses intervention in midfoot arthritis			Activity limitation 7			Conclusion positive
				Effect Size (ES) Total 0.4 Pain 0.6 Activity limitation 0.4			MDC and ES findings are significant
FFI-R; Rao, S [76] 2010	A measure of clinical importance of orthoses intervention		FFI-R long 68 items	MDC Total 5, Pain 5 Stiffness 6, Disanility 7, Activity limitation 7 Psychosocial 7 ES: Total 0.7, Pain 0.84, Stiffness 0.31, Disability 0.6, Limitation 0.57, Psycho social 0.32			N 30 Mid foot pain
							Conclusion positive

**Table 7:** Studies using foot function measures in surgical interventions

**Instrument**	**1**^**st **^**Author**	**Objective**	**Population *(N, Sex, Age, Dx, location)***	**Analysis**	**Items/Domains/Subscales**	**Response type**	**Summary evaluation**
Foot Function Index (FFI), 2000	Lin, S [39]	Validation of AOFAS forefoot outcomes of arthrodesis surgery	N: 16 Mean age: 44 (SD=13.96) 8 male	Pre-post surgery	FFI	VAS	Both FFI and AOFAS scores were improved at post surgery.
			Dx: Tarsometatarso injury/degenerative arthritis	Follow-up 36 months (24–65 months)	23 items		Conclusion: useful
			Location: USA	FFI and AOFAS were applied at pre-surgery and at follow up	3 domains		
FFI, 2002	Watson, TS [61]	Validation with VAS pain scale with SF-36 short form in plantar fasciotomy	Group I N (control): 75 Mean age: 46 (range: 20–78) 14 male	Retrospective observational Follow up duration 26.4 months	FFI	VAS	FFI scores were improved.
			Group II N (surgery): 46 Mean age: 46 (Range: 25–78) 9 male	Group II filled out FFI and SF-26 at post-surgery only	23 items		FFI scores reflected activities of daily living. SF-36 s cores reflection satisfaction of physical and role model.
			Dx: Sub-Calcaneal pain syndrome	Validation with VAS pain scale SF-36 short form	3 domains		Conclusion: useful.
			Location: USA				
FFI, 2003	Mulcahy, D [56]	Surgery-Reconstruction of the forefoot; FFI scores were used to test if there was correlation with WOMAC, AOFAS HMIP, and AOFAS LMIP.	N: 79 14 male Mean age: 59 (Range: 24–80)	Retrospective observational; Follow up 6yrs.+3 mo (6mo-19 years)	FFI; 23 items; 3 domains	VAS	FFI pain subscale was used to monitor pain in both groups.
			Dx: RA forefoot deformity				Conclusion: useful
			Mean age of surgery: 52 years (range: 23–79)				
			Group 1 stable 1^st^ ray. (no surgery)				
			Group 2: 1^st^ ray surgery				
			Location: Canada				
FFI, 2004	Ibrahim T [48]	Surgery- MTP joint replacement; Validation of AOFAS Hallux scale scores with FFI scores from those who did not have surgery and those who had surgery	N: 8, 1 male	Retrospective observational; Follow up for 17 months	FFI	VAS	Correlation observed between the scores of AOFAS and FFI
			Mean age: 58 (Range: 51–80)		23 items		Note: AOFAS Hallux scale had not been validated.
			Dx. Hallux rigidus		3 domains		Conclusion: useful
			Location: UK				
FFI, 2004	Vallier, HA [52]	Surgery-Open reduction internal fixation; Correlation of FFI and musculoskeletal function assessment (MFA)	N: 100 60 male	Retrospective observational	FFI	VAS	Scores of FFI and MFA were correlated
			Mean age: 32.6 (Range: 13–77)	Follow up 36 months (12–74 months)	23 items		Conclusion: useful
			Dx: Talar neck fracture	FFI was applied to N=59 at follow-up	3 domains		
			Location: USA				
FFI, 2005	Taranow, WS [49]	Surgery- metalic hemiarthroplasty: Do FFI scores improve at post-operation	N: 28 17 male	Retrospective observational case review	FFI	VAS	FFI scores from pre to post operation showed significant improvement.
			Mean age: 52.9 (Range: 38–71)	Follow 33.4 months	23 items		Conclusion: useful
				(3–mo-111mo)			
			Dx: Hallux rigidus		3 domains		
			Location: USA				
FFI, 2005	Grondal, L [40]	Surgery-Athrodesis vs. Mayo resection of MTP; FFI scores as outcomes	N: 31; 26 male	RCT not-blinded, ANOVA and multiple comparisons	FFI	VAS	FFI scores at post-surgery within groups were improved and there no significant differences between the groups.
			Mean age: 54 yrs		23 items		Conclusion: useful
			(Range: 33–77)				
			Resection N=: 16		3 domains		
			Fusion N=: 15				
			Dx: RA painful forefoot deformity				
			Location: Sweden				
FFI, 2005	Daniels, TR [62]	Surgery -Free tibular graft; FFI scores were validated with MODEMS and SF-36 short form	N: 28, 13 male	Observational	FFI 21 items (2 items about orthoses were not applicable) 3 domains	Likert	The scores of FFI, SF-36 and MODEMS were demonstrating similar improved outcomes at post-surgery
			Mean age: 52 (Range: 22–76)	Follow-up: 36 months (26–52 months)			Conclusion: useful
			Dx: Vascularized fibular bone graft	FFI was applied at pre-surgery and at 6 and between 26–54 month post surgery			
			Location: Canada				
FFI, 2005	Lee, S [63]	Surgery -Isolated sesamoidectomy; FFI disability sub-scale validated with VAS pain scale and SF-36 short form	N; 32; 8 male	Retrospective observational	FFI 9 items	VAS	The scores of FFI disability and VAS pain sub-scales were correlated.Conclusion: useful
			Mean age: 37.2 (Range: 18–65)	62 month	1 domain: disability scale		
				Post-op N=: 20			
			Dx: Hallux alignment				
			Location: USA				
FFI, 2006	SooHoo, NF [64]	Surgery- Any type of foot and ankle surgery; Validating AOFAS, SF-36 and measuring Standard Response Mean (SRM) and effect size (ES)	N: 25; 6 Male	Pre-post surgery FFI was applied at pre-surgery and 6 months post-surgery	FFI	VAS	Of the instruments used, scores of the pain subscale was the only measure reflecting high SRM (−0.83) and ES (−0.86). Therefore, pain is the most important outcome in studies regarding chronic foot and ankle pain.
			Mean age: 40 (Range: 21–69)		23 items		Conclusion: useful
			Dx: Chronic foot and ankle conditions requiring surgery		3 domains		
			Location: USA				
FFI, 2006	Van der Krans, A [41]	Surgery- Calcaneal Cuboid arthrodesis; Correlation with AOFAS clinical rating index (CRI) of the hind foot	N: 20; 4 Male	Pre-post surgery	FFI-Dutch 15 items	5-point verbal scale	FFI and CRI scores showed significant improvements
			Mean age: 55 (Range: 30–66)	Follow-up 25 months (13–39 months)	Pain and function subscales		Conclusion: useful
			Dx: Flat foot	FFI was applied at pre-surgery and ad follow-up			
			Location: Netherlands				
FFI, 2006	Harris, M [53]	Surgery- High impact fracture repair; Correlation with Musculoskeletal function assessment (MFA)	N: 76; 45 Male	Pre-post surgery follow up 26 months (24–38 months). FFI was applied at pre-surgery, 6 and 12 weeks and at 6 months by mail, telephone, and was self-administered.	FFI	VAS	High FFI score occurred in those with the worse fractures and external fixation. This is also reflected in MFA scores.
			Mean age: 45 (Range: 17–81)		23 items		Conclusion: useful
			Dx: distal tibial plafond fracture		3 domains		
			Location: USA				
FFI, 2006	Stegman M [42]	Surgery-Triple arthrodesis; Correlation with AOFAS hind foot scores	N: 81; 38 Male	Pre-post surgery	FFI Dutch	Likert	FFI-5pt and AOFAS hind foot scores improved 89%. However, patient did not perceive the benefit of the procedure.
			Mean age: 40.5 (Range: 14–79)	FFI applied at pre-surgery and 1 yr (1–4) post surgery	15 items		Conclusion: useful
			Dx: Hind foot disorders		2 domains		
			Location: Netherlands				
FFI, 2007	Jung, HG [45]	Surgery-Fusion of tarso metatarso-joint; Correlation with SF-36, AOFAS	N: 67; 12 Male	Retrospective observational	FFI	VAS	Scores of the FFI, SF-36 AOFAS and VAS pain scale were markedly improved at post-surgery
			Mean age: 60.2 (Range: 35–84)	Follow for 40.6 months	23 items		Conclusion: useful
			Dx: Non-traumatic osteoarthritis of the tarso-meta-tarso joints	FFI applied at post-surgery	3 domains		
			Location: USA				
FFI, 2008	Vesely, R [43]	Surgery – Tibio Calcaneal arthrodesis; Correlation with ankle-hind foot score	N: 20; 16 Male	Retrospective observational	FFI	VAS	The scores of FFI and ankle hind foot were improved.
			Mean age: 58.7 (Range: 23–72)	FFI applied at post-surgery, time unknown	23 items		Conclusion: useful
			Dx: Traumatic arthritis of the ankles	Article in Czech with English abstract.	3 domains		
			Location: Czech Republic				
FFI, 2008	Stropek, S [37]	Surgery- arthroscopy	N: 26; 6 Male	Pre-post surgery observational	FFI	VAS	FFI pain scale scores were markedly improved at post surgery in 79% of the patients
			Age: male: 45; female: 49	FFI applied at pre-surgery and at 3 month follow-up	Pain scale		Conclusion: useful
			Dx: Calcaneal spur		9 items		
			Location: Czech Republic				
FFI, 2008	Schutte, BG [50]	Surgery-Total ankle replacement; pain and function outcome measure	N: 47; 16 Males	Pre-post surgery	FFI-Dutch	Likert	Total scores improved at post–surgery
			Mean age: 57.1 (range 37–81)	FFI applied at pre-surgery and at follow up	18 items		Conclusion: useful
			Dx: Ankle joint deformity	Duration of follow up 28 months (range 12–67)	Pain and difficulty subscales		
			Location: Netherlands				
FFI, 2008	Ward, CM [57]	Surgery-Reconstruction; Validation of SF 26 with FFI	N: 25; 14 Male	Pre-post surgery	FFI	VAS	At follow up the FFI scores were in the mid-range. The scores for smokers were worse than non-smokers, females were worse than males. FFI activity limitation and disability scores were correlated with SF-36 physical component scores.
							
			Mean age: 15 (Range: 8.7-25)	FFI applied at mean age of 41.5 years after 26.1 yrs follow-up	23 items		Conclusion: useful
			Dx: Flexible Cavovarus Charcot Marie-Tooth		3 domains		
			Location: USA				
FFI, 2009	Castellani, C [65]	Surgery-Fixation with cannulation osteosynthesis; Outcomes of an intervention	N: 21; 11 Male	Retrospective observational	FFI	VAS	At follow-up 3 of the 21 (14%) had poor FFI disability scores
			Dx: Transitional fracture of distal tibia	FFI was applied at 3.8 yrs after implants removal	23 items		Conclusion: useful
			Age 13.7 (1.4)		3 domains		
			Location: Austria				
FFI, 2009	Bonnin, MP [51]	Surgery – Total ankle arthoplasty; Correlations of FAAM (foot and ankle ability measure)	N: 140; 50 Male	Pre-post surgery pre at pre-surgery FAAM and FFI was applied, and also at 53.8 ±29 months (12–125) post- surgery	FFI	VAS	FFI pain scores were no different between OA and RA groups. The FFI scores were improved and were similar to that of FAAM.
			Mean age: 60.9 (Range: 26–90)		23 items		Conclusion: useful
			Dx: OA: 100 RA: 40		3 domains		
			Location: France				
FFI, 2009	Potter, MQ [54]	Surgery- Intraarticular fracture of the Calcaneus; Correlation with AOFAS hind foot scores	N: 73; 52 Male	Retrospective observational FFI applied at follow up of 12.8 years (5–18.5)	FFI	VAS	Scored of FFI, AOFAS hind foot and Calcaneal scores were correlated.
			Dx: Calcaneal fracture		23 items		Conclusion: useful
			Location: USA		3 domains		
FFI, 2010	Aurich, M [66]	Surgery-Arthroscopic chondrocyte implant; Correlation with AOFAS hind foot scores and Core Scale of the foot and ankle module of the Academy of Orthopedic Surgeon (AAOS)	N: 18; 13 Male	Retrospective observational FFI was applied at pre-arthroscopy and at follow-up, with mean duration of 19 months	FFI 18 items; Pain and function subscales	Likert	FFI scores improved comparable with those of AOFAS results and Core Scale scores
			Mean age: 29.2 (SD 10.2 years)				Limitation: Use of FFI measures with caution in individual whose. functional level is better than the level of activities of daily living.
			Dx: Osteochondral lesion of talus/tibia				Conclusion: useful
			Location:Australia				
FFI, 2010	Van der Heide, HJL [59]	Surgery-Correction pes cavo varus; Validation AOFAS lesser toe module	N: 39; 6 Male	Pre-post surgery; FFI applied at pre-surgery and 40 month post-surgery	FFI-Dutch	VAS	FFI pain and function scores improved post-surgery
			Mean age: 59 (Range: 29–81)				Conclusion: useful
			Dx: RA lesser MTP		23 items		
			Location: Netherlands		3domains		
FFI- Dutch, 2010	Kroon, M [60]	Surgery-Correction pes cavo varus; Validation AOFAS hind foot scale	N: 15; 8 Male	Pre-post surgery FFI applied at pre and 50 month post surgery	FFI-Dutch	Likert	Pain and function scores improved post surgery
			Mean age:40 (SD 14)		18 items		Conclusion: useful
			Dx: Cavo varus foot deformity		Pain and function subscales		
			Location: The Netherlands				
FFI, 2010	Van Doeselaar, DJ [46]	Surgery-Fusion of MTP; Correlation with VAS pain and satisfaction	N: 62	Pre-post surgery; FFI applied at pre-surgery and 12 month post-surgery	FFI Dutch; 18 items	Likert	FFI-5 pts scores were improved.
			2 groups				
			Dx: H rigidus; N: 27; 9 Male				Conclusion: useful
			Mean age: 58 (Range: 42–72)				
			Dx: H valgus; N: 35; 6 Male				
			Mean age: 61 (Range: 37–76)				
			Location: Netherlands				
FFI, 2010	Doets, HC [44]	Surgery-Salvage arthrodesis for failed TAA; Correlating with AOFAS and VAS pain scale	N: 18; 4 Male	Retrospective observational FFI applied at follow up, 3–12 years	FFI-Dutch	5 point rating scale	FFI scores improved similar to that of AOFAS, VAS pain, disability and satisfaction measure
			Mean age: 55 (Range: 27–76)		15 items		Conclusion: useful
			Dx: Failed TAA		Pain and function subscales		
			Location: Netherlands				
FFI, 2010	Niki, H [47]	Surgery-TMT fusion and osteotomy; Concurrent assessment of FFI and SF-36 and Japanese Society for Surgery of the Foot and Ankle Score	N: 30; 1 Male	Pre-post surgery FFI was applied at pre-surgery and at 36 mos follow-up	FFI	VAS	The scores of all instruments were improved at post-surgery.
			Mean age: 53.6 (Range: 45–67)		23 items		Conclusion: useful
			Dx: RA fore-foot deformity		3 domains		
			Location: Japan				
FFI, 2010	Schlegel, UJ [58]	Surgery-Club foot correctional; Post-surgery foot health assessment	N: 98; 72 Male	Retrospective observational FFI was applied at 8.2 years (0–11.2); Post surgery N: 46 (50%)	FFI	VAS	FFI scores indicated good foot health.
			Mean follow-up: 4.5M (Range: 1–68)		23 items		Conclusion: useful
			Dx: Club foot		3 domains		
			Location: Germany				
FFI, 2010	Gaskill, T [55]	Surgery- Internal fixation of the instraarticular Calcaneal fracture; Concurrent evaluation with OAFAS hind foot	N: 146; 99 Male	Retrospective observational FFI was applied at post-surgery 8.98 years	FFI	VAS	FFI scores of Group 1 were better than Group 2 at post surgery.
			Group 1 <50 yrs; N: 99; 65 male		23 items		Conclusion: useful
			Mean age: 36 (Age range)		3 domains		
			Group 2 >50 years; N: 47; 33 male				
			Mean age: 58 (Range: 50–84)				
			Dx: Calcaneal fracture				
			Location: USA				
FFI, 2010	Eberl, R [67]	Surgery- Various surgical techniques were applied; Post surgery outcomes	N: 24; 18 Male	Retrospective observational	FFI	VAS	FFI scores improved in both groups. Group 1 scored better than Group 2.
			Mean age: 13.2 (Range: 5–17 yrs)	Follow-up 3.2 years (7 months-8.2 years)	23 items		Limitation: The author stated that use of self-report in instrument in children may result in spurious outcomes, due to their pronounced potential for compensation.
			Group 1 <12 years; N: 9; Age : 9.2	FFI applied at follow up	3 domains		Conclusion: useful
			Group 2 >12 years; N: 15; Age: 14.6				
			Dx: Complex ankle injuries				
			Location: Australia				

**Table 8:** Studies using foot function measures in orthotic intervention

**Instrument**	**1**^**st **^**Author**	**Study and objective**	**Population *(N, Sex, Age, Dx, location)***	**Methods & Analyses**	**Items/Domains/ Subscales**	**Measurement scale**	**Summary evaluation**
FFI,1995	Budiman-Mak, E [74]	Outcome measure of orthotic intervention in hallux valgus deformity	N=102	RCT double blind Intent to Treat Analysis FFI applied at baseline and each follow up visit	FFI	VAS	This study suggest that foot orthosis can prevent or slowed the progression of hallux valgus deformity
			Treatment group (N: 52)		23 items		
			Mean age: 60.2 (SD 10.6)		3 domains		
			Male: 46 (88.5%)				
			Control group (N:50)				
			Mean age: 58.8 (SD 11.9)				
			Male: 43 (86%)				
			DX:RA				
			Location: USA				
FFI, 1996	Conrad, KJ [70]	Outcome measure-Pain and function measures	N:102	RCT double blind Post –test Random effect model for longitudinal data	FFI	VAS	This study showed no benefit on pain and disability measures between treatment group and placebo group
			Treatment group (N: 52)Mean age: 60.2 (SD 10) 46 male	FFI applied at baseline and at each follow up visit	23 items		Conclusion: useful
			Control group (N:50) Mean age: 58.8 (SD11.9) 43 male		3 domains		
			Dx: RA				
			Location: USA				
FFI, 1997	Caselli, MA [77]	Outcome measure - Effectiveness of the intervention	N: 34; Mean age: 43 (28–59) 12 male	RCT, not-blinded FFI was applied at baseline and at 4 weeks	FFI	Categorical rating scale	58% (11/19) of participants showed improvement in pain scores Conclusion: useful
			Group 1: Group with magnet (N: 19)		23 items		
			Group 2: Group with no magnet (N: 15)		3 domains		
			Dx: Heel pain				
			Location: USA				
FFI, 1997	Caselli, MA [68]	Outcome measure -Effectiveness of the intervention	N: 35; Mean age: 42 (23–65); 18 male	RCT not blinded FFI was applied at baseline and at 4 weeks	FFI	Categorical rating scale	FFI scores improved at 4 weeks reported as the following:
			Group 1: Viscoped (N: 16)		23 items		60% (Group1)
			Group 2: Poron (N: 12)		3 domains		43% (Group 2)
			Group 3: Control(N: 7)				10% (Group 3)
			Dx: Painful submetatarsal hyperkeratosis				Conclusion: useful
			Location: USA				
FFI, 1999	Pfeffer, G [78]	Outcome measure – primary interest is in pain subscale outcome at 8 weeks	N: 236; Mean age: 47 (23–81); 160 male	RCT not blinded 6 months interventions multi-centers. FFI was applied at baseline and at 8 week intervals At 8 weeks the group response rate was 88.2%	FFI	VAS rating scale	Pain subscale scores improved at 8 weeks
			Group 1: Stretching only (N: 39) Mean age: 47 (25–81) 11 male		23 items		Pain change scores controlled for covariates. Results are reported as the following:
			Group 2: Custom orthoses & stretch (N: 34) Mean age: 48.5 (23–69) 11 male		3 domains		Group 1: -17.2
			Group 3: Silicon & stretch (N: 51) Mean age: 49.5 (30–75) 17 male				Group 2: -16.9
			Group 4: Rubber & stretch (N: 43) Mean age: 44 (27–69) 11 male				Group 3: -23.9
			Group 5: Felt & stretch (N:42) Mean age: 48 (26–76) 13 male				Group 4: -24.5
			Dx: Proximal plantar fasciitis				Group 5: -20.2
			Location: USA				Conclusion: useful
FFI, 2001	Slattery, M [82]	Outcome measure – effectiveness of the intervention	N: 46; Mean age: 24 (6.2) Sex not reported	Observational 6 weeks FFI applied at baseline	FFI	VAS rating scale	FFI scores of pain and disability subscales markedly improved at 6 weeks
			Dx: Hemophilic foot and ankle arthropathy at level 1–5 joint damange		23items		Conclusion: useful
			Location: Australia		3 domains		
FFI, 2002	Gross, MT [79]	Outcome measure – Effectiveness of the intervention correlation with 100 meter walk and VAS pain scale	N: 15; 8 male	Pre-post test design FFI was applied at baseline and post orthosis at 12–17 days	FFI 18 items Pain and disability scales	VAS rating scale	Pain and disability improved. The author suggested to modify FFI items if FFI will be used for plantar fasciitis.
			Mean age male: 43.8 (SD=6.3)				Conclusion: useful
			Mean age female: 45.9 (SD=11.9)				
			Dx: Plantar fasciitis				
			Location: USA				
FFI, 2002	Woodburn, J [80]	Outcome measure – effectiveness of the intervention	N: 98; Orthosis/vsControl	RCT double blind; 30 months study. FFI was applied at 3, 6, 12, 18, 24, and 30 months	FFI	VAS rating scale	FFI scores improved at the completion of the RCT
			Orthosis (N: 50) Mean age: 54 (SD=11.8) 16 male		23 items		Conclusion: useful
			Control (N: 48) Mean age: 53 (SD=11.1) 17 male		3 domains		
			Dx: RA rear foot valgus deformity				
			Location: UK				
FFI, 2005	Powell, M [83]	Outcome measure – Validation of The Pediatric Pain VAS Questionnaires, Pediatric quality of life (PedQOL) inventory physical function scale	N: 40; Custom orthoses: N: 15; 2 Male Mean age: 12.14	RCT 3 arms, Single blinded	FFI	VAS rating scale	The largest improvement of FFI scores was in the custom orthoses. VAS scoring appears applicable in children
			Insert N: 12; 4 Male Mean age: 12.7	Intent to Treat Analysis; ANOVA	23 items		Conclusion: useful
			Athletic shoes N: 13; 4 Male Mean age: 13.77	FFI was applied at baseline and at 3 months	3 domains		
			Dx: JRA and foot pain				
			Location: USA				
FFI, 2006	Magalahaes, E [69]	Outcome measure – Concurrent measure with Health Assessment Questionnaires (HAQ)	N: 36; 5 Male	Prospective observational	FFI	VAS rating scale	FFI scores in pain, disability, activity limitation improved; no correlations with HAQ scores
			Orthosis N: 28	2 treatment groups; 6 months trial	23 items		Conclusion: useful
			Sham N: 8	FFI was applied at baseline, 30, 90, and 180 days	3 domains		
			Mean age: 46 (32–68) RA years 11 (1–34)				
			Location: Brazil				
FFI, 2007	Williams, AE [71]	Outcome measure – Concurrent measure with FHSQ for designed shoes intervention	N: 80; 35 male Age: N/A	RCT single blinded; 12 weeks trial. FFI was applied at baseline and 12 weeks N:34 completed the study	FFI	VAS rating scale	Both scores of FFI and FHSQ were improved at 12 weeks
			Group 1: Designed shoes (N: 40); 11 male		23 items		Between groups general health was unchanged
			Group 2: Traditional shoes (N: 40) 19 male		3 domains		Conclusion: useful
			RA 17 years (14.4 yrs)				
			Dx: Hallux valgus				
			Location: UK				
FFI, 2008	Lin, JL [81]	Outcome measure – Validation with AOFAS VAS pain scale SF-36	N: 32; 6 male	Observational 7–10 years (mean 8.8 years); FFI was applied at the end of the observation			FFI scores for pain and disability were improved and well correlated with AOFAS scores
			Dx: Stage II posterior tibialis tendon dysfunction (PTTD)				Conclusion: useful
			Location: USA				
FFI, 2009	Cho, NS [72]	Outcome measure – Validation with VAS pain scale	N: 42; Semi-rigid insole: N: 22	RCT single blinded 6 month trial FFI was applied at baseline and 6 month At 6 months N34 completed the study	FFI	VAS rating scale	Semi-rigid insole group showed markedly improved FFI scores
			0 male				
			11fore foot/11 hind foot		23 items		Conclusion: useful
			Mean age: 48.7 (SD=11.6)		3 domains		
			Soft insole: N: 20; 0 male 11 fore/10 hind foot				
			Mean age: 48.7 (SD=11.7)				
			Dx: RA foot deformity, hind or forefoot				
			Location: Korea				
FFI, 2009	Novak, P [84]	Outcome measure – Correlation with 6 minute walk time	N: 40; Mean age: 56.23; 2 male	RCT double blinded 6 months trial FFI was applied at baseline visits 1, 2, and 3 at 6 months	FFI	VAS rating scale	Pain improved correlation with 6 minute walk time was moderate
			Orthosis (N: 20) Mean age: 55.7 (SD=9.31) RA: 10.5 yrs (SD=8.17)		9 items		Conclusion: useful
			Control (N: 20) Mean age: 56.75 (SD=11.1) RA: 11.5 yrs (SD=6.86)		Pain scale		
			Dx: RA				
			Location: Slovenia				
FFI, 2009	Baldassin, V [35]	Outcome measure – pain relief	N: 142; Custom Orthosis: N=72	RCT double blind; 8 weeks trial. FFI was applied at 4 and 8 weeks	FFI	VAS rating scale	Less pain was observed in both groups but no significant differences between groups
			Mean age: 55.7 (SD=12.4)		23 items		Conclusion: useful
			RA: 47.2 yrs (SD=8.17) 51 male		3 domains		
			Prefabricated orthosis: N=70		Pain subscales 9 items (modified)		
			Mean age: 47.5 (SD=11.5)				
			Dx: Plantar fasciitis				
			Location: Brazil				
FFI-R, 2009	Rao, S [75]	Outcome measure – FFI-R scores translated to clinical measure MDC_90_, Correlation with medial mid-foot pressure loading	N: 20; 0 male	Intervention 4 weeks FFI-R was applied at pre and post intervention Statistician was blinded from data sources	FFI-R	Likert	Total FFI-R scores improved correlated with significant reduction in pressure loading of the medial aspect of the midfoot
			Mean age: 63 (55–78)		68 items		Conclusion: useful
			Full length orthosis		Long form		
			Dx: Midfoot arthritis				
			Location: USA				
FFI-R, 2010	Rao, S [76]	Outcome measure – Clinical measure MDC 90 validation with segmental foot kinematic values	N: 30; 2 male	Intervention 4 weeks FFI-R was applied at pre and post intervention	FFI-R	Likert	Full length foot orthoses reduced motion of the 1^st^ metatarsophalangeal and was significantly correlated with FFI-R scores
			Mean age: 62 (47–78)		68 items		Conclusion: useful
			Full length carbon graphite orthosis		Long form		
			Dx: Midfoot arthritis				
			Location: USA				
FFI, 2010	Welsh, BJ [73]	Outcome measure – validation with foot kinematic values VAS pain scale	N: 32; 6 male	Case series 24 weeks Pre-post test design	FFI	VAS rating scale	FFI pain subscale significantly improved and met the criteria of equivalence to analgesic response. This pain reduction was not correlated with that of the biomechanical changes of the 1^st^ metatarsophalangeal joint.
			Mean age: 42 (SD=11.5)		9 items		Conclusion: useful
			Pre-fabricated vs. custom orthosis		Pain subscale		
			Dx: MTP joint pain				
			Location: UK				
FFI, 2010	Clark H [85]	Outcome measure – Orthosis reduced pain and disability and correlated with gait parameter	N: 41; Gender not reported	RCT single blind 16 weeks trial. FFI was applied at baseline, 8 and 16 weeks	FFI	VAS rating scale	FFI scores were improved in orthoses and simple insole groups but the intervention did not improve gait parameter.
			Orthosis: N: 20; Simple insole: N: 21		23 items		Conclusion: useful
			Age>18 years; RA>3 years		3 domains		
			Location: New Zealand				

**Table 9:** Studies using foot function measures in various interventions

**Instrument**	**1**^**st **^**Author**	**Objective**	**Population *(N, Sex, Age, Dx, location)***	**Analysis**	**Items/domains/subscales**	**Response type**	**Summary evaluation**
Foot Function Index, 2005	Cui, Q [86]	Improvement in pain and function	N: 5; Mean age: 40 (range: 25–54); 3 male	Retrospective study; Follow-up 24 months (16–30 months). FFI was applied at pre and at post treatment	FFI	VAS	FFI scores improved on 3 out of 5 patients post surgery.
		Cortisone injection and arthroscopic surgery	Dx: Post traumatic ankle adhesive capsulitis		Pain and disability subscales		Conclusion: useful
			Location: USA		18 items		
Foot Function Index, 2005	Di Giovanni, BF [87]	Reduction of foot pain Stretching exercise and wearing foot insert	N: 101; 33 male	Randomized clinical Trial Longitudinal mixed-model analysis of covariance FFI was applied at baseline and at 8 weeks (N=:82, A=46, B=36). At 2 years (N:=66, A=39,B=27)	FFI	VAS	FFI pain scores improved at 2 weeks and much improved at 2 years
			Mean age: 45 (range 23–60)		Pain subscale		Group A had a better scores than B
			Group A: Plantar fascia stretching		9 items		Conclusion: useful
			Group B: Achillus tendon stretching				
			DX: Plantar fasciitis				
			Location: USA				
Foot Function Index, 2009	Kulig,K [88]	Validation of physical activity scale (PAS) and 5 minutes walk test, and simple heel raise test.	N=: 10; Gender: NA	Exercise intervention: 10 weeks Follow up: 6 months. FFI was applied at baseline, 10 weeks and 6 months	FFI	VAS	FFI pain and function subscales were used to monitor pre- and post-intervention outcomes.
			Mean age:52.1 (SD6.5)		23 items		Conclusion: useful
			DX: Posterior tibial tendon dysfunction		3 domains		
			Location: USA				
Foot Function Index, 2010	Rompe, JD [89]	Outcomes: Change scores between observations. Stretching and shock wave therapy	N=54; 18 male	Randomized parallel treatment 15 months trial. Intend to treat analysis FFI was applied at baseline, 4 and 15 months	FFI	VAS	FFI pain scores were better in stretching exercise group
			Mean age: 53.1 (SD =27.7)		Pain subscale		Conclusion: useful
			Dx: Plantar Fasciotomy		9 items		
			Location: Germany				

**Table 10:** Studies using foot function measures in observational studies

**Instrument**	**1**^**st **^**Author**	**Study and objective**	**Population *(N, Sex, Age, Dx, location)***	**methods & analyses**	**Items/domains/subscales**	**Response type**	**Summary evaluation**
FFI, 2004	Novak, P [4]	Epidemiology of Type II Diabetes Mellitus					
		Correlation of pain score with 6 minute walk time; Comparing intergroup pain score	Total N: 90; 3 groups;	Cross-Sectional study	FFI	VAS scale	High pain score correlated with shorter distance walk, group with Type II diabetes neuropathy with symptoms showed the highest pain scores
				Descriptive & correlation statistics			
			Neuropathy with symptoms N: 30 Mean age 64.87 (SD=11)		9 items		Conclusion: useful
			20 male		Pain scale		
			Neuropathy, no symptoms N:30; Mean age: 64.87 (SD=11)				
			20 male;				
			Healthy volunteers N: 30; Mean age: 64.87 (SD=11)				
			20 male;				
			Slovenia				
FFI, 2004	Williams, AE [90]	Epidemiology Rheumatic diseases	N: 139; 39 male	Cross sectional study	FFI	VAS scale	FFI scores showed a high prevalence of foot and ankle pathologies, which indicated the need of podiatry care
				Descriptive statistics			
		To assess foot health status	Age: NA		23 items		Conclusion: useful
			Inflammatory and degenerative joint diseases		3 domains		
			UK				
FFI, 2006	Williams, AE [91]	Epidemiology of Paget diseases of the foot Concurrent measures of FSI and quality of Life 12-items short form	N: 134; 64 male	Cross sectional study Descriptive statistics	FFI	VAS scale	Correlations of scores were not found between instruments
			Mean age: 74.5 (46–91)		23 items		Conclusion: not useful
			UK		3 domains		
FFI, 2006	Rosenbaum, D [95]	Plantar sensitivity assesstment	N:25; 2 male	Observational study	FFI 23 items 3 domains	VAS scale	FFI was to evaluate foot sensation related to RA
		Rheumatoid arthritis foot	Mean age: 55 (SD=9.9) RA; 9.6 (SD=7)				Conclusion: useful
		Evaluate the correlation of painful walking and loss of sensitivity of the plantar surface of the foot	Germany				
FFI, 2008	Schmeigel, A [96]	Pedobarography in rheumatoid arthritis	N: 112; Mean age: 55 (SD=11)	Observational	FFI	VAS scale	Higher FFI scores correlated with pedograph scores
		To evaluate the function and pedographic impairment	RA1; N: 36; HAQ scores 0–1		23 items; 3 domains		Conclusion: useful
		Correlation of foot pain and pedograph	3 male; Mean age: 50.6 (SD=10.5)		RA1: FFI total score: 20.7 (SD=12.9)		
			RA2; N: 38 HAQ scores 1.1-2		RA2: FFI total score: 28.8 (SD=12.1)		
			1 male; Mean age: 55.2 (SD=10.4)		RA3: FFI total score: 48.7 (SD=15.9)		
			RA3 N: 38 HAQ scores 2.1-3				
			2 male; Mean age: 58.5 (SD=11.3)		Control NA		
			Control N:20 Mean age: 53.2 (SD=12.3)				
			Germany				
FFI, 2010	Kamanli, A [92]	Foot Bone Mineral Density	RA: N: 50; RA<3 yrs 1 male, 5 female	Cross sectional study	FFI	VAS	Moderate-strong correlation of FFI scores with disease duration, VAS pain scale, Stoke index, HAQ, femur bone mineral density (BMD). No correlation with foot BMD.
		To assess the correlation of FFI scores with VAS pain scale, HAQ Ritchie articular index, and stoke index		Descriptive statistics	Pain scale 9 items		
			RA>3 yrs				Conclusion: useful.
			4 male, 40 female				
			Mean age: 52 (SD=10.9)				
			OA: N:40; 3 male				
			Mean age: 52.4 (SD=11.8)				
			Healthy volunteers; N: 14				
							
			Turkey				
FFI, 2010	Goldstein, CL [94]	Foot and ankle trauma	N: 52; 31 male	Cross sectional study the mean duration post trauma 15.5 months (1 month-10 years)	FFI	VAS	There was a high correlation among FFI scores and the 5 listed instruments.
		Correlation of FFI, SF-12, SMFA, FAAM, AAOS, AOFAS	Mean age: 43.3 (18–85)		9 items		Conclusion: useful
			OA; Foot and ankle trauma		Pain scale		
			Canada				
FFI, 2010	Kavlak, Y [93]	Elderly men Concurrent measure with VAS pain scale, foot problem score, hind foot function scale	N: 53; 53 male	Cross sectional study	FFI	VAS scale	FFI was simple and comprehensive and was significantly correlated with hind foot function scale, and scores of timed up and go.
			Mean age: 73.8 (7.08)		23 items		Conclusion: useful
			Foot problems		3 domains		
			Turkey				

### Objective 3. Enumerate the strengths and weaknesses of the FFI and FFI-R as reported in the literature

This was a qualitative summary of the results as found in Table 5 and Table 6.

### Objective 4. Improving the FFI-R metrics

Table 11 summarizes results of the Rasch analysis. This was a reanalysis of the FFI-R database collected in 2002 with the aim of improving FFI-R metrics.

**Table 11:** Reliability and unidimensionality of the full scale, short form, and subscales

	**Full scale**	**Short form**	**1-11**	**12-19**	**20-39**	**40-49**	**50-68**
	**(68 items)**	**(34 items)**	**(Pain)**	**(Stiffness)**	**(Difficulty)**	**(Limitation)**	**(Social issues)**
**Person Reliability**	.96	.95	.89	.89	.94	.78	.84
**Cronbach’s Alpha**	.98	.97	.93	.95	.97	.87	.94
**Unidimensionality Criteria** (Ratio of the raw variance explained by measures: Unexplained variance in 1^st^ contrast ≥ 3)	56.8/10.6=	60.2/15.8=	66.7/22.1=	67.5/34.7=	72.7/15.5=	63.4/19.2=	53.6/18.1=
	5.4	3.8	3.0	1.94^1^	4.69	3.3^2^	2.96^3^
	Yes	Yes	Yes	No	Yes	Yes	No

^1^ Further inspection of the data revealed that the two-factor solution was associated with the severity of the items, where the two factors were actually low and high severity stiffness, i.e. opposite poles of the same factor. Therefore, the scale is useful as a measure of stiffness. ^2^ These were the results after removing item 41 (ASSISTO).

^3^ Approximately unidimensional.

### Descriptive analysis methods

Quantitative data were reported using simple statistics expressed as the sum, means, and standard deviations for continuous variables and as frequencies for categorical data. (Tables 1, 2, 3, and 4) Analytic statements and evaluations/comments for each article collected are summarized in Table 12. This depicts the summary of FFI and FFI-R uses as illustrated in Objective 2, and in six tables (Tables 5, 6, 7, 8, 9 and 10).

**Table 12:** Summary of FFI and FFI-R uses as provided in detail in Tables 5-10

**FFI/FFI-R instrument usage**	**Category**	**Name of instrument**	**First Author’s name [reference number]**
Measurement			
(Details in Tables 5 &6)	A) New Instrument	FFI	Budiman- Mak E [7]
		FFI-R	Budiman-Mak E [11]
		FFI-site to site	Saag KG [23]
		AOS	Domsic RT [24]
		FFI Likert Scale	Agel J [25]
	B) FFI as Criterion Validity	HAQ and SFC	Bal A [26]
		SF-36	SooHoo NF [27]
		Navicular joint alignment	Shrader JA [28]
		FIS	Helliwell P [29]
		WOMAC and DAS 44	Van der Linden M [30]
		AOFAS	Lau JT [31]
		AOFAS Hallux	Baumhauer JF [32]
		AOFAS	Ibrahim T [33]
	C) Cultural adaptation/Translation	Dutch-FFI-5pts	Kuyvenhoven MM [3]
		FFI-G	Naal FD [34]
		FFI-Taiwan Chinese	Wu SH [36]
		FFI- Spanish	MAPI Institute [38]
Surgeries			
(Details in Table 7)	a) Arthrodeses and Fusions	FFI, FFI-Dutch,	Lin SS [39], Grondal L [40],van der Krans A [41], Stegman M [42], Vesely R [43], Doets HC [44], Jung HG [45], van Doeselaar DJ [46], Niki H [47]
	b) Arthroplasty	FFI, FFI pain and difficulty subscales,	Ibrahim T [48], Taranow WS [49], Schutte BG [50], Bonnin MP [51]
	c) Fracture Care	FFI	Vallier HA [52], Harris AM [53], Potter MQ [54], Gaskill T [55]
	d) Reconstruction Surgery	FFI, FFI-Dutch	Mulcahy D [56], Ward CM [57], Schlegel UJ [58], van der Heide HJ [59], Kroon M [60]
	e) Other surgery	FFI, FFI disability subscale, FFI pain subscale, FFI pain and disability subscales	Watson TS [61], Daniels TR [62], Lee S [63], SooHoo NF [64], Stropek S [37], Castellani C [65], Aurich M [66], Eberl R [67].
Orthoses			
(Details in Table 8)	a) Forefoot	FFI	Caselli MA [68], de P Magalahaes [69], Conrad KJ [70], William AE [71], Cho NS [72], Welsh BJ [73], Budiman-Mak E [74].
	b) Mid foot	FFI-R	Rao S [75], Rao S [76]
	c) Hind foot	FFI, FFI, Brazil (pain subscale modified),	Caselli MA [77], Baldassin V [35], Pfeffer G [78], Gross MT [79], Woodburn J [80],Lin JL [81], Slattery M [82], Powell M [83], Novak P [84], Clark H [85]
Other interventions			
(Details in Table 9)	Injection	FFI pain and disability subscales	Cui Q [86]
	Stretching exercise	FFI, FFI pain subscale	DiGiovani BF [87], Kulig K [88], Rompe JD [89].
Observational studies			
(Details in Table 10)	Foot morbidities		
	In diabetes mellitus	FFI pain subscale	Novak P [4]
	In rheumatic diseases	FFI	Williams AE [90], Williams AE [91]
	In bone mineral density	FFI pain subscale	Kamanli A [92]
	In elderly	FFI	Kavlak Y [93]
	In foot post-injury	FFI pain subscale	Goldstein CL [94]
	In rheumatoid arthritis	FFI	Rosenbaum D [95], Schmeigel A [96]

### Rasch analysis method

To address specific critiques of the FFI-R found in the literature, the unidimensionality of the FFI-R and its subscales were evaluated against the Rasch model. The statistical package Winsteps version 3.72.3 [14] was used to conduct a principal components analysis (PCA) of the standardized residuals to determine whether substantial subdimensions existed within the items [15-17] and whether the FFI-R L, the FFI-R S, and the 5 subscales were unidimensional. The criterion used to define unidimensionality was a large variance (> 40%) explained by the measurement dimension [18]. Unexplained variance in the first contrast of the data should be small and fall under the criterion of 15% for a rival factor. We chose a ratio of variance of at least 3 to 1 in the first principal component [19], compared to the variance of the first component of residuals.

#### Rasch reliability statistics

Reliability was estimated with Cronbach’s Alpha and Rasch person reliability statistics. Both indices reflect the proportion of variance of the person scores or measures to total variance (i.e., including measurement error). Unlike Cronbach’s Alpha, Rasch person reliability is based on the estimated locations of persons along the measurement continuum, excluding those with measures reflecting extreme (zero or perfect) scores and including cases with missing data. For both indices, our criterion for acceptability was .80.

#### Response category analysis

One requirement of the Rasch model is monotonicity: the requirement that, as person ability increases, the item step response function increases monotonically [20]. This means that choosing one categorical response over the prior—for example, moving from selecting “2 = A little of the time,” to selecting, “3 = Most of the time,”—increases with person ability. The proper functioning of the rating scale is examined using fit statistics, where: (i) outfit mean squares should be less than 2.0, (ii) average measures advance monotonically with each category, and (iii) step calibrations increase monotonically [21,22].

## Results

### Review of the literature

**Figure 1:**
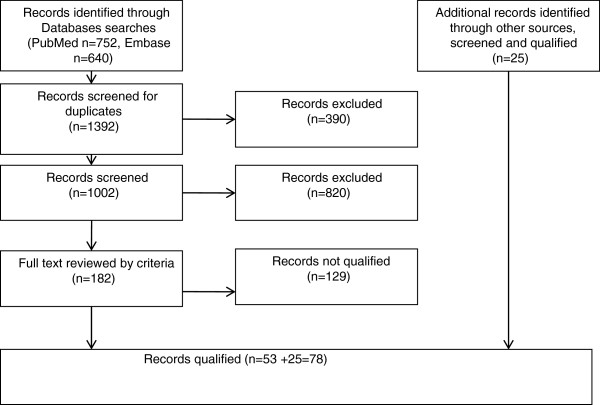
Algorithm of searched and screened for qualified paper.

Articles were obtained by using the search method defined in the Methods section; the search results included 752 articles from PubMed/MEDLINE and 640 articles from Embase. Further screening and selection procedures, as detailed in Figure 1, yielded 182 full-text articles. Of these, 53 articles were qualified for review. Twenty-five more articles were obtained from the search engine BioMedLib and from manual searches. A total of 78 articles qualified for this review, summarized and categorized into several tables,

### Objective 1: Assessment of the prevalence of the FFI or FFI-R usage, population characteristics, and study locations

Among the 78 studies, we identified 4714 study participants for whom the FFI or FFI-R instrument had been used to measure foot health. This sample consisted of 1914 (41%) male participants and 2688 (57%) female participants, with a mean age of 48.58 years (SD, 4.9 years). There was a discrepancy of 2% between the sums of male and female participants, because gender was not reported in three studies (Table 1). Most of the participants were individuals and young adults, and a few studies involved juvenile participants. The types of studies included measurement practice studies (n=17), surgery studies (n=30), studies of orthotics (n=19) or other clinical interventions (n=4), and observational studies (n=8). We identified 20 different diagnoses of foot and ankle pathology that were measured by FFI and FFI-R (Table 2). Among them, RA and plantar fasciitis were the two most common diagnoses and were also noted to be the most painful and disabling foot conditions. These studies were conducted by investigators in 17 countries; the United States, the Netherlands, and the United Kingdom were the three most frequent users of the FFI and FFI-R in studies involving foot and ankle problems (Table 3).

Table 4 displays the versatility of the FFI with all 3 domains and FFI Subscales and FFI-R uses across the studies. This shows that clinicians and researchers were choosing the FFI scales depending on the nature of their studies. Among the various scales of the FFI, we found the FFI with all 3 domains (full scale), the FFI pain subscale only, and a combination of the pain and disability subscales to be the most frequently used, whereas the FFI-R was the least frequently used. The Dutch adaptation of the FFI, the FFI-5pts, was mostly used in the Netherlands as an outcome measure in studies of many surgical interventions.

In summary, the FFI with all 3 domains, or as subscales, was frequently chosen as a measurement instrument across various studies and countries and among various age groups and sexes, for the assessment of acute and chronic foot and ankle conditions.

### Objective 2: Uses of the FFI and FFI-R in the field of foot health research

The uses of the FFI and FFI-R are provided in detail in Tables 5, 6, 7, 8, 9, and 10. Table 12 describes the study types, the name of the instruments, and the first author’s name and the reference number. The studies are grouped by how the instruments were used and ordered chronologically within group.

#### Measurement, validation and cultural adaptation

Table 12 describes the utility of the FFI and FFI-R in studies of foot function measures and includes 17 articles. *Category A New Instruments*. Includes four articles in which foot health measures are described including the original FFI [7], the FFI-R [11]. The FFI Side to Side was derived from pain and disability subscales of the FFI [23]. The Ankle Osteoarthritis Scale (AOS) [24]; measured foot problems related to foot and ankle osteoarthritis. Agel et al. [25] modified the rating scale of the FFI pain and function subscales from the visual analog rating scale (VAS) to the Likert categorical scale; this modification was tested in a sample of individuals with non-traumatic foot complaints, and the metric of the Likert scale was valid. *Category B FFI as Criterion Validity*. Articles in this category describe several health measures and use the FFI full scale or subscales to validate these measures. Bal et al. [26] found a strong correlation of FFI scores and scores of RA functional measures: the Health Assessment Questionnaire (HAQ) and Steinbrocker Functional Class (SFC). SooHoo et al. [27] found that the Rand 36-Item Short Form Health Survey (SF-36) scores of a sample of individuals with foot and ankle disorders were moderately correlated with FFI scores and concluded that FFI scores can be used to monitor the quality of life of these patients. Shrader et al. [28] measured the stability of navicular joint alignment and found that this measure correlated well with the FFI scores of the sample. Helliwell et al. [29] developed a new measure, the Foot Impact Scale (FIS), to measure the impact of foot problems on foot health in a sample of individuals with RA; the metric of FIS was validated with the FFI and HAQ. In an RA study, van der Leeden et al. [30] reported that Western Ontario and McMaster Universities Osteoarthritis Index (WOMAC) and Disease Activity Scores in 44 joints (DAS 44) were correlated with FFI scores; furthermore, this author discerns the correlations that the FFI pain subscale scores correlated with forefoot pain while the FFI function subscale scores correlated with hindfoot problems. The FFI scores were also used as validation measures of the American Orthopedic Foot and Ankle Society (AOFAS) clinical rating scales, an instrument that was widely used by foot and ankle surgeons [31]. These validation studies were reported by Baumhauer et al. [32] for the AOFAS hallux clinical rating scale and by Ibrahim et al. [33] for the AOFAS clinical rating scale, which was well to moderately correlated with FFI scores. The latter finding was based on his study with a 41% response rate in a sample consisting of 45 individuals. *Category C Cultural Adaptation or Translation*. The first translation of the FFI was the Dutch-language instrument known as Dutch FFI-5pts [3]. The German-language translation of the instrument is the FFI-G [34]; the FFI was also translated into Brazilian Portuguese [35], Taiwan Chinese [36], Turkish [26], and Czech [37]. There was also a Spanish translation conducted by the MAPI Institute in Lyon, France [38]. These translations complied with rigorous language translation procedures; occasionally, some item adjustments of the scales were needed. In summary, the FFI was developed with good reliability and validity; it also inspired and served as criterion validity for newer foot health measures and attracted the attention of researchers around the world, who conducted translations and adaptations of the tool into their native languages and cultures.

Table 6 is a supplement to Table 5 and displays the clinimetrics of the instruments listed in Table 5; measures were metrically good, with reliability and validity values greater than 0.7 with one exception where the pain subscale had a reliability of 0.64 [3].

#### Surgical intervention

The FFI is one of the outcome measures most frequently used by AOFAS members [31]. It was first used to measure surgical outcomes. The surgical interventions and outcomes are summarized in Table 7. There are 30 articles, categorized generally according to type and location of surgical procedure. Five distinct procedural categories were identified as follows: (a) arthrodeses within the foot or ankle [39-47], (b) arthroplasty within the foot or ankle [48-51], (c) fracture care of the foot or ankle [52-55], (d) deformity reconstruction surgery of the foot or ankle [56-60], and (e) various surgical interventions for chronic conditions [61-64]. The FFI was also used to assess outcomes of less invasive procedures, such as calcaneal spur treatment by arthroscopy [37], distal tibia repair using fixation with cannulation osteosyntheses [65], arthroscopic chondrocyte implant of the tibia and fibula [66], and surgical interventions for complex ankle injuries [67]. In summary, the FFI and the Dutch FFI-5pts appeared to be useful in measuring outcomes of various surgical procedures in children, adults, and individuals with acute, chronic, and congenital foot and ankle problems.

#### Orthotic interventions

Table 8 lists studies using foot function outcome measures in orthotic interventions in the foot and ankle. The studies assessed the impact of orthotic treatment on forefoot, midfoot, and hindfoot/ankle pathology. Orthotic treatment on the forefoot in patients with RA improved the scores for pain, disability and activities [68,69], however the scores were unchanged in the study by Conrad et al. [70]. Other studies using special shoes and shoe inserts showed symptoms of relief in hallux valgus pain [71] hindfoot and forefoot problems [72,73]; and slowing the progression of hallux valgus in early RA [74]. Midfoot studies assessing the treatment of full length orthoses on pain relief [75], and mobility were performed using the FFI-R as an outcome measures [76]. For hindfoot conditions treatment with orthoses included studies of heel pain [77], plantar fasciitis [35,78,79], stabilizing hindfoot valgus [80], correction of posterior tibialis tendon dysfunction [81], destructive hemophilic arthropathy of the foot and ankle [82] and juvenile idiopathic arthritis of the foot and ankle [83]. Shoes/shoe inserts have also been found to relieve foot and ankle pain from arthritides [84,85]. In summary, the FFI and FFI-R clearly provided useful outcome measures for orthotic management of a wide range of foot and ankle disorders.

#### Medical intervention

The FFI also was used to measure foot health outcomes associated with medical interventions (Table 9), such as cortisone injection of the ankle adhesive capsulitis [86]; the injection resulted in improved FFI pain and disability subscale scores. Di Giovanni et al. [87] measured the outcome of stretching exercises for plantar fasciitis versus Achilles tendonitis; both groups showed improvement in FFI pain subscale scores. Kulig et al. [88] used the FFI pain and disability subscales to measure the outcomes of exercise intervention in posterior tibial tendon dysfunction. Rompe et al. [89] reported the FFI pain score improved in the stretching treatment group of a randomized clinical trial using stretching and shockwave therapy to treat patients with plantar fasciopathy. Overall, the FFI was useful in measuring the outcomes of conservative interventions in chronic foot and ankle conditions.

#### Observational studies

Investigators had chosen the FFI scores or the subscale scores to determine the prevalence and disease burden of foot and ankle conditions in the general population (Table 10). Novak et al. [4] used FFI scores to evaluate type 2 diabetes with and without neuropathy and identified that group with neuropathy had worse FFI scores. Williams and Bowden [90] correlated high FFI scores to foot morbidity in rheumatic diseases, and estimated cost of care/staffing concerns for that patient subset. Williams [91] also used the FFI scores in patients with Paget’s disease and noted the impacts on plantar foot pressures, gaits, and ambulation abilities. Kamanli et al. [92] correlated the scores of the FFI and foot bone mineral density, then extrapolated these scores to that individual’s skeletal bone density. Kavlak and Demitras [93] reported a strong correlation of FFI scores with the scores of VAS pain scale, foot pain scale (FPS), and hindfoot function scale (HFS) in patients with foot problems. Goldstein et al. [94] noted that FFI scores of individuals with previous foot injuries had a high correlation with 6 other foot function instruments. Rosenbaum et al. [95] found that plantar sensory impairment of the foot in patients with RA was correlated with poor FFI scores. Schmiegel et al. [96] found that pedobarograph scores of patients with RA with foot pain were correlated with poor FFI and HAQ scores. In summary, FFI scores were useful in detecting the prevalence of foot and ankle problems and as a measure of concurrent validity for other foot health measures in various chronic foot conditions.

In all, we found the FFI instrument was frequently chosen as an outcome measure of surgical, orthotic, and medical treatments, but its application was wider than we originally imagined. It was not limited to outcome measures; FFI scores were also applied in the promotion of foot health as a common public health issue and in increasing the awareness of health system administrators. The FFI was also used in the validation of newly developed foot health measures.

### Objective 3: The strengths and weaknesses of the FFI and FFI-R as reported in the literature

*FFI*: The FFI questionnaire had good psychometric properties [97-100], and the pain subscale was sensitive to change during instrument development [13]. In a study about treatment of plantar fasciitis in individuals with chronic foot pain, SooHoo et al. [64] reported that the pain subscale of the FFI had high standard response mean (SRM) and high effect size (ES) as outcome measures of surgery in chronic foot and ankle problems. While Landorf and Radford measured the clinical ability to detect a change as minimal important difference (MID) in plantar fasciitis [101]. All these clinical measures add to the credibility of the FFI as a self-reporting measure, the FFI reflects patients’ assessment of their symptoms/health status, which directs providers about proper care planning and progress toward treatment goals. FFI is one of the most cited measures of its kind [102].

There are weaknesses of the FFI. During the development of the index, clinicians generated the questionnaire items without patient participation [13,97]; therefore, items might not fully reflect patients’ needs, might be sex biased [7], and might not be applicable to high-functioning individuals. A theoretical model was not part of the design, nor were the items related to footwear [13,103], which are essential to support the construct of this instrument. It is also lacking items for measuring quality of health and satisfaction with care; however, these items can be appended as a global statement in the questionnaire. In all, the FFI has been the most studied and widely used foot-specific self-reporting measure; however, further testing by gender, age, race, language, etc. would provide assurance of its generalizability.

*FFI-R****:*** The FFI-R was developed in response to criticism of the FFI and to address issues of contemporary interest. Most original items from the FFI were selected in the development of FFI-R, and new items about footwear and psychosocial factors were added, which improved its construct coverage. Patients and clinicians were involved in the generation of items. Its design closely followed the ICF theoretical model [13]; its psychometric properties are strong and are based on the IRT 1-parameter or the Rasch measurement model. It was designed to be a comprehensive measure of foot health–related quality of life, with both long and short forms [99], allowing clinicians and researchers to choose the measures they need for the intended study. Although the FFI-R did not include information on clinical ability to measure change in its development, Rao et al. [75,76] did measure the minimal detectible change (MDC) and the effect size, in individuals with midfoot arthritis, which also added to the credibility of its metrics.

### Objective 4: The newly analyzed FFI-R with improved psychometric values

#### The full scale and short form

For the FFI-R L (68 items) [11], person reliability was high: 0.96, respectively. In the PCA, 56.8% of the variance was explained by the measure, with only 10.6% of the variance explained by the first factor of residuals. These findings support that the full FFI-R meets the unidimensionality requirement of the Rasch model. Further, the criterion for unidimensionality was a ratio of the raw variance in the first contrast of residuals that was 5.4 (i.e., greater than 3). For the FFI-R S (34 items) [11], person reliability was 0.95, similar to the reliability estimates of the FFI-R L. The PCA of the FFI-R S revealed that unidimensionality criteria were also satisfied. This supports the use of a short form of the measure, because the item response burden on patients is lower, at 34 questions. Because this measure is as reliable as the full measure, its use is supported for clinical settings.

#### Subscales

All subscales of the FFI-R had strong person reliability estimates (Table 11), ranging from 0.78 to 0.94 for person reliability. The PCA indicated that unidimensionality held for each subscale, with the exception of the stiffness subscale. Further inspection of the data revealed that the two-factor solution reflected groups of the low-severity and high-severity items and was not the result of a competing factor. Unidimensionality for the limitation subscale was met after dropping item 41 (ASSISTO), an item listed in the FFI-R database. Overall, the subscales of the FFI-R satisfied unidimensionality criteria and were reliable measures of the latent traits (Table 11).

#### Response category analysis

The response category analyses for each of the subscales (done after collapsing Categories 5 and 6) revealed that, for the first three subscales (pain, stiffness, and difficulty), the response categories behaved as required by the Rasch model. However, for the subscales of limitation and social issues (both of which are time scales), there was some indication that respondents had difficulty distinguishing between, “2 = A little of the time,” and, “3 = Some of the time.” We considered, then, collapsing these categories and making all FFI-R subscales have four possible response categories. This would ensure uniformity of the measure and decrease the burden on patient response. Therefore, the first three subscales, which measure severity, “3 = Severe pain,” “4 = Very severe pain,” and “5 = Worst pain imaginable,” were collapsed. This was justified because all three captured the notion of severe pain. Overall, the analyses showed that the response to each item functioned well with the four-item response categories.

## Discussion

This review evaluated 78 eligible articles (Figure 1). In the past 20 years, it appears that the FFI and FFI-R were widely used across national and international clinical and research communities. The instruments were administered to over 4700 study participants of males and females worldwide, across age groups, with 20 different diagnoses consisting of congenital, inflammatory/degenerative, acute and chronic foot and ankle problems. The FFI was also incorporated into other newer foot health measures [23,24], and also underwent changes in the measurement scale from VAS to Likert scale such as the one conducted by Agel et al. [25]. The scale changes also occurred in FFI adaptation to the Dutch [3], German [34], and Taiwanese Chinese [36] including our revised FFI-R [11] to give a few examples. The strong metrics of FFI subscales and full scale (Table 12, Category A), facilitated the investigator’s choice to use its subscale(s) or full scale in clinical or research applications as appropriate. The FFI was also frequently used as validation criterion for other foot health measures (Table 12, Category B); this validation usage has elevated the credibility of the FFI as an outcome measure for foot and ankle problems. Since the FFI was developed using CTT procedures, it is sample and content dependent, therefore its metrics were tested in many different samples, where its metrics were proven to be consistently strong. The exception was in the study of Baumhauer et al. [32] where high foot functioning was evident in the sample; therefore, investigators should exercise caution in the interpretation of this result. While the FFI was developed initially as disease specific for early RA, in later years, it was used in many non-RA foot and ankle problems and was proven to be a valid measure as well. The FFI and FFI-R were frequently used as outcome measures in surgical and clinical interventions with positive results (Tables 7, 8, 9, and 10). The FFI scores were also used in many observational studies (Table 10) and those reports might be helpful for researchers and the health system administrators in establishing a health policy. Although the FFI was extensively studied and generally received positive ratings [23,29,102], we realized the need for improvement in the measures of FFI and FFI-R and have discussed this issues comprehensively under Objective 3 in this paper. We conducted a re-analysis and made improvements to the metrics and scales of FFI-R as presented in Table 11 and questionnaires FFI-R Long Form (See Additional file 1), and Short Form (See Additional file 2).

In recent articles about FFI used as outcome measures, the authors have included the clinical measures; the effect size, and standard response mean [64], and minimal important difference [101], while Rao et al. reports minimal detectible change and effect size of the FFI-R [75], all these have increased the credibility of the clinical use of the FFI to help in power analysis and sample size estimation for future studies.

### Limitations of this review

Our literature search was limited to publications written in the English language and covered only publications until 2010; therefore, this might exclude the FFI- and FFI-R–related published articles not written in English, as well as those more recent articles published in English.

## Conclusions

The FFI pioneered measuring outcomes in foot health. This instrument has been tested through time and adapted in its measures as it was frequently used in full scales or subscales to measure outcomes in various clinical practice or research studies. The FFI has also had a role in shifting the paradigm from a reliance on physical and biochemical findings as outcomes to the use of outcomes that are relevant to patients. Thus, the measure established patient-centered, valid, reliable, and responsive hard data endpoints. The rating scales also underwent changes; for practicality and user-friendliness in clinical and research settings. The FFI was recognized as a valid instrument and used as a validation criterion of other measures. It was adapted and translated into multiple languages. It was applied to all age groups, across genders and was useful in measuring varied medical and surgical conditions.

In realizing the scope of FFI applications, we acknowledge the contributions of friends and colleagues around the world who not only used the FFI in their studies but also made adaptations and translations to make the FFI a versatile instrument in promoting and maintaining foot health. The FFI-R has good psychometric properties and is available in long and short forms for ease of clinical use. In response to findings in this review, we conducted a rigorous analysis to strengthen the metrics of the FFI-R and changed the rating scales to be more user-friendly and practical.

Both the FFI and FFI-R are in the public domain and permission to use them is free of charge. They are available from the developers of these instruments and from the AOFAS web site. These instruments are self-administered and are written at an eighth-grade reading level. The FFI scores are interpreted as 0%-100% for each subscale and the overall score. Higher FFI and FFI-R scores indicate poor foot health and poor foot health-related quality of life. The FFI and FFI-R put minimal burden on respondents and the questionnaires are not emotionally sensitive. The administrative burden is also minimal and it does not require formal training to score or to interpret [104]. Translations and adaptations are available in Dutch [3], Taiwan Chinese [36], German [34], Turkish [26], Brazilian Portuguese [35], and Spanish [38].

This review attests to the widespread use of foot health measures, and we have noticed the advancement of foot health in general across diagnoses. It has been a privilege for us to serve patients, clinicians, and researchers to fulfill the mission in improving foot health through the use of the FFI and FFI-R. These instruments are available for users, and can be downloaded as they are presented as electronic files.

## Abbreviations

AOFAS: American Orthopedic Foot and Ankle Society; CTT: Classical test theory; EMBASE: Excerpta Medica Database; FFI: Foot Function Index; FFI-R: Foot Function Index Revised; EBM: Elly Budiman-Mak; FFI-R L: Foot Function Index Revised Long Form; FFI-R S: Foot Function Index Revised Short Form; HAQ: Health Assessment Questionnaire; IRT: item response theory; JM: Jessica Massa; KJC: Kendon J Conrad; Medline: Medical Literature Analysis and Retrieval System; PUBMED: public Medline; RA: rheumatoid arthritis; RMS: Rodney M. Stuck; VAS: visual analog rating scale; AAOS: American Academy of Orthopedic Surgeon; ANOVA: Analysis of Variance; AOS: Ankle Osteoarthritis Index; BMD: Bone Mineral Density; CA: Crohnbach’s Alpha; CRI: Clinical Rating Index; CV: Calcaneal Varus; DAS 44: Disease Activity Score in 44 joints of patient with rheumatoid arthritis (RA); DX: Diagnosis; EF: External Fixation Procedure; ES: Effect Size; FAAM: Foot and Ankle Ability Measure; FFI-5pts: Dutch Foot Function Index with 5 point Likert Scale; FFI-G: Foot Function Index - German Language; FHSQ: Foot Health Status Questionnaire; FIS: Foot Impact Scale; FPS: Foot Problem Score; FSI: Foot Structure Index; FX: Fracture; HFS: Hind Foot Function Scale; HMIP: Hallux Metatarso-interphalangeal Joint; HR: Hallux Rigidus; ICC: Interclass Correlation Coefficient; JIA: Juvenile Idiopathic Arthritis; JRA: Juvenile Rheumatoid Arthritis; LMIP: Lesser Metatarso-interphalangeal Joint; MCS: Mental Component Score of SF-36; MDC: Minimal Detectible Change; MFA: Musculoskeletal Function Assessment; MFDQ: Manchester Foot Disability Questionnaires; MID: Minimal Important Difference; MODEMS: Musculo-skeletal Outcome Data Evaluation and Management System; MTP: Metatarsophalangeal Joint; NA: Not Applicable; OA: Osteoarthritis; PAS: Physical Activity Scale; PCS: Physical Component Score of SF-36; PedQL: Pediatric Quality of Life Scale; PF: Plantar Fasciitis; PTTD: Posterior Tibialis Tendon Dysfunction; QOL -12: Quality of Life 12 items short form; RAI: Ritchie Articular Index; RCT: Randomized Control Trial; SD: Standard Deviation; SF-36: Rand 36 items health survey form; SF-36 MCS: Mental Component Score of SF-36; SF-36 PCS: Physical Component Score of SF-36; SF-12: Rand 12 items short form health survey; SFC: Steinbrocker Functional Class; SMFA: Musculoskeletal Function Assessment; SRM: Standard Response Mean; SI: Stroke Index; TAA: Total Ankle Arthroplasty; TMT: Tarso Meta-metatarso Joint; UCLA: University of California - Los Angeles; WOMAC: Western Ontario MacMaster University Osteo Arthritis Index.

## Competing interests

The authors declare that they have no competing interests.

## Authors’ contributions

EBM, KJC, have contributed in drawing the concept and design of this paper, EBM initiated the literature search, reviewed, scrutinized them, and collected the abstracts and organized into tables. KJC, RMS and JM reviewed the tables and all authors participated in drafting the manuscript. KJC and JM also reanalyzed the original FFI-R data and revised the subscales and FFI-R response categories. All authors participated in revising the manuscript and have given final approval of the version to be published.

## Supplementary Material

Additional file 1 Revised FOOT FUNCTION INDEX (FFI-R). Click here for file

Additional file 2 Revised FOOT FUNCTION INDEX (FFI-R) Short Form. Click here for file
