# Supplementary material for: A review of the foot function index and the foot function index – revised
Source: J Foot Ankle Res. 2013 Feb 1;6:5. doi: 10.1186/1757-1146-6-5 (PMC3579714; doi:10.1186/1757-1146-6-5)
Supplement: Additional file 2 — Revised FOOT FUNCTION INDEX (FFI-R) Short Form. [file 1757-1146-6-5-S2.docx]

**Revised FOOT FUNCTION INDEX (FFI-R) Short Form**

Subject ID: [_____]_____]_____]______] [Date: [___]___] / [___]___] / [___]___]___]

# PAIN

**Please read before answering.**

- Please circle the number that indicates how bad your foot pain was in each of the following situations during the past week.
- For example, when asked how severe your foot pain was at its worst, if you feel “No pain,” circle the number 1 and if you felt “Severe pain,” circle the number 4.
- If, for some items, the question does not apply, circle the number 5.
- Please provide an answer for every item.

1. DURING THE PAST WEEK, HOW SEVERE WAS YOUR FOOT PAIN:

No Mild Moderate Severe

Pain pain pain pain

1. Before you get up in the morning? . . . . . . . . . . . . 1 2 3 4

2. When you first stood without shoes? . . . . . . . . . 1 2 3 4

3. When you stood wearing shoes? . . . . . . . . . . . . . . 1 2 3 4

4 When you walked wearing shoes? . . . . . . . . . .. . . 1 2 3 4

5. When you stood wearing custom shoe inserts? . . 1 2 3 4 5 = do not use

inserts

6. When you walked wearing custom shoe inserts? . 1 2 3 4 5= do not use

inserts

7. At the end of a typical day? . . . . . . . . . . . . . . . . . 1 2 3 4

Subject ID: [_____]_____]_____]______]

**STIFFNESS**

**Please read before answering.**

- Please circle the number that indicates how bad your foot stiffness was in each of the following situations during the past week.
- For example, when asked how severe your foot stiffness was at its worst, if you feel “No stiffness,” circle the number 1 and if you felt “Severe stiffness,” circle the number 4.
- If, for some items, the question does not apply, circle the number 5.
- Please provide an answer for every item.

1. DURING THE PAST WEEK, HOW SEVERE WAS YOUR FOOT STIFFNESS:

No Mild Moderate Severe

stiffness stiffness stiffness stiffness

8. Before you get up in the morning? . . . . . . . . . . . . 1 2 3 4

9. When you stood without shoes? . . . . . . . . . . . . . 1 2 3 4

10. When you walked without shoes? . . . . . . . . . . . 1 2 3 4

11. When you stood wearing shoes? . . . . . . . . . . . . . . 1 2 3 4

12. When you walked wearing shoes? . . . . . . . . . .. . . 1 2 3 4

13. When you walked wearing custom shoe inserts? . 1 2 3 4

14. Before you went to sleep at night? . . . . . . . . . . . 1 2 3 4

Subject ID: [_____]_____]_____]______]

# DIFFICULTY

**Please read before answering.**

- Please circle the number that indicates how much difficulty you had performing each activity because of your foot problems during the

past week.

- For example, when asked how much difficulty your foot problems caused when walking around the house, if you had “No difficulty,” circle the number 1 and if it was ” Severe difficulty,” circle the number 4.
- If, for some items, the question does not apply, circle the number 5.
- Please provide an answer for every item.

2. DURING THE PAST WEEK, HOW MUCH DIFFICULTY DID YOUR FOOT PROBLEMS CAUSE YOU:

No Mild Moderate Severe difficulty difficulty difficulty difficulty

15. Walking outside on uneven ground? . . . . . . 1 2 3 4

16. Walking four or more blocks? . . . . . . . . . . . 1 2 3 4

17. Climbing stairs? . . . . . . . . . . . . . . . . . . . . . . 1 2 3 4

18. Descending stairs? . . . . . . . . . . . . . . . . . . . . 1 2 3 4

19. Standing on tip toes? . . . . . . . . . . . . . . . . . . 1 2 3 4

20. When you carried or lifted objects

weighing more than five pounds? . . . . . . . . 1 2 3 4

21. Getting out of a chair? . . . . . . . . . . . . . . . . . 1 2 3 4

22. Walking fast? . . . . . . . . . . . . . . . . . . . . . . . 1 2 3 4

Subject ID: [_____]_____]_____]______]

3. (cont.) DURING THE PAST WEEK, HOW MUCH DIFFICULTY DID YOUR FOOT PROBLEMS CAUSE YOU:

No Mild Moderate Severe difficulty difficulty difficulty difficulty

23. Running? . . . . . . . . . . . . . . . . . . . . . . . . . . . 1 2 3 4

24. Keeping your balance? . . . . . . . . . . . . . . . . 1 2 3 4

25. Walking with assistive devices? . . . . . . . . 1 2 3 4

Subject ID: [_____]_____]_____]______]

# ACTIVITY LIMITATION

**Please read before answering.**

- Please circle the number that indicates how often you performed each of these activities in the past week because of your feet.
- For example, when asked how often you used a cane indoors because of foot problems, if you used one “None of the time,” circle the number 1 and if you used one “All of the time,” circle the number 4.
- If, for some items, the question does not apply, circle the number 5.
- Please provide an answer for every item.

4. DURING THE PAST WEEK, HOW MUCH OF THE TIME DID YOU:

None Some Most All

of the time of the time of the time of the time

26. Stay indoors most of the day because of

foot problems? . . . . . . . . . . . . . . . . . . . . . . . . . . 1 2 3 4

27. Limit your outdoor activities because of

foot problems? . . . . . . . . . . . . . . . . . . . . . . . . . 1 2 3 4 5= No outdoor

activities

28. Limit your leisure/sport activities

because of foot problems? . . . . . . . . . . . . . . . 1 2 3 4 5 = Do not play

sports

Subject ID: [_____]_____]_____]______]

# SOCIAL ISSUES

**Please read before answering.**

- Please circle the number that indicates how often you experienced the following feelings in the past week because of your feet.
- For example, when asked how often you felt a fear of falling because of foot problems, if you felt fear “None of the time,” circle the number 1 and if you felt fear “All of the time,” circle the number 4.
- If, for some items, the question does not apply, circle the number 5.
- Please provide an answer for every item.

5. DURING THE PAST WEEK, HOW MUCH OF THE TIME DID YOU EXPERIENCE:

None of Some Most All of

the time the time the time the time

29. Embarrassment due to footwear? . . . . . . . . . . . 1 2 3 4

30. Feeling awful because of foot problem? . . . . . . 1 2 3 4

31. Limit social activities due to foot problems? . . 1 2 3 4

32. Difficulty participating in social activities

due to footwear? . . . . . . . . . . . . . . . . . . . . . . . 1 2 3 4 ______

33. Burden of taking medication to control

foot pain? . . . . . . . . . . . . . . . . . . . . . . . . . . . . 1 2 3 4 ______

34. Concern about limited work around the house?. 1 2 3 4 ______

**SUBJECT COMMENTS:**

Please comment about:

1. Were the directions clear?
2. Were any of the questions difficult to understand?
3. Were any of the questions unclear? If yes, which ones and why?
4. Did any of the questions make you uncomfortable? If yes, which ones and why?
5. Are there any issues about your feet that were not asked or that you would add to the questionnaire? If yes, which issues?
6. Did you have any problems with this questionnaire that you would like to mention? If yes, which problems?

Thank you for participating in this study.

Pain score: ______

Stiffness score: ______

Difficulty score: ______

Activity score: ______

Social score: ______

Cumulative score: ______
